# Heterogeneity of a dwarf phenotype in Dutch traditional chicken breeds revealed by genomic analyses

**DOI:** 10.1111/eva.13183

**Published:** 2021-01-19

**Authors:** Zhou Wu, Chiara Bortoluzzi, Martijn F. L. Derks, Langqing Liu, Mirte Bosse, Sipke Joost Hiemstra, Martien A. M. Groenen, Richard P. M. A. Crooijmans

**Affiliations:** ^1^ Wageningen University & Research, Animal Breeding and Genomics Wageningen The Netherlands; ^2^ Centre for Genetic Resources, the Netherlands (CGN) of Wageningen University & Research Wageningen The Netherlands

**Keywords:** bantam, chicken, convergent selection, dwarfism, genome‐wide association study, heterogeneity, whole‐genome sequencing

## Abstract

The growth of animals is a complex trait, in chicken resulting in a diverse variety of forms, caused by a heterogeneous genetic basis. Bantam chicken, known as an exquisite form of dwarfism, has been used for crossbreeding to create corresponding dwarf counterparts for native fowls in the Dutch populations. Here, we demonstrate the heterogeneity of the bantam trait in Dutch chickens and reveal the underlying genetic causes, using whole‐genome sequence data from matching pairs of bantam and normal‐sized breeds. During the bantam‐oriented crossbreeding, various bantam origins were used to introduce the bantam phenotype, and three major bantam sources were identified and clustered. The genome‐wide association studies revealed multiple genetic variants and genes associated with bantam phenotype, including *HMGA2* and *PRDM16*, genes involved in body growth and stature. The comparison of associated variants among studies illustrated differences related to divergent bantam origins, suggesting a clear heterogeneity among bantam breeds. We show that in neo‐bantam breeds, the bantam‐related regions underwent a strong haplotype introgression from the bantam source, outcompeting haplotypes from the normal‐sized counterpart. The bantam heterogeneity is further confirmed by the presence of multiple haplotypes comprising associated alleles, which suggests the selection of the bantam phenotype is likely subject to a convergent direction across populations. Our study demonstrates that the diverse history of human‐mediated crossbreeding has contributed to the complexity and heterogeneity of the bantam phenotype.

## 1 INTRODUCTION

Human activities influence the demographic history and phenotypic diversity of animals through artificial selection and crossbreeding (Andersson, 2001; Bruford et al., 2003). In the course of domestication, the human‐mediated selection was a major force that drove the phenotypic differentiation, which has been further enhanced by the breed formation during recent centuries (Andersson & Georges, 2004). The selection of body size in animals has been a major focus during domestication (Andersson & Georges, 2004; Mignon‐Grasteau et al., 2005; Zeder, 2012). Albeit the rapid growth and the large body size are of great interest to commercial breeders, attention for small‐sized animals has been raised as well.

Dwarf animals are characterized by a short body stature (Boegheim et al., 2017). Despite the general description of varying growth reduction, comprehensive phenotypic heterogeneity has been shown for dwarfism. A clear example is the extreme stature variations across dog breeds (Hulsegge et al., 2013; Parker et al., 2009; Sutter et al., 2007). The dwarf phenotype in chickens is observed and measured by different narratives, including but not limited to body weight, height, and shank length (Boegheim et al., 2017; Cole, 2000). Three distinct types of dwarfism have been described in chickens based on the physiological and genetic properties: sex‐linked dwarfism, which is well studied and caused by mutations in the growth hormone receptor gene (*GHR*) (Agarwal et al., 1994; Burnside et al., 1992); autosomal dwarfism (adw) that is associated with a nonsense mutation in the transmembrane protein 263 gene (*TMEM263*) (Wu et al., 2018); and third the bantam phenotype, for which the genetic cause is unknown. The origin of the word “bantam” refers to a region in south‐east Asia (Java island) and came to be known as a form of small fowl of exquisite appearance that became prevalent in Europe.

Compared to other typical dwarfisms, bantam is unique in the way it shows the reduction in body weight, yet no clear other malformations and no evidence of affected viability. Bantams in the Netherlands usually have around 50–60% reduced body weight compared to their corresponding counterparts (Figure S1A,B, Appendix S1). Traditional bantam breeds or true bantams are known for their existence without a large form counterpart. Examples of true bantams from the Netherlands are the Dutch bantam, the Dutch booted bantam, and the Eikenburger bantam (Verhoef & Rijs, 2014). Nowadays, the bantam chicken has become an important component of Dutch chicken breeds, which encompasses a recent history of human‐mediated crossbreeding of the bantam phenotype. Neo‐bantam chickens are the hybrid bantam counterparts produced by this crossbreeding procedure with the goal of miniaturizing, which is called bantamization (Bortoluzzi et al., 2018). The creation of the neo‐bantams is based on crossing the normal‐sized indigenous breeds with existing bantam breeds, followed by repeated backcrossing with the normal‐sized indigenous breeds and selecting for the bantam phenotype. In other words, the normal‐sized Dutch breeds have been artificially crossed with existing bantams, which resulted in neo‐bantams that represent the dwarf stature but meanwhile keep similar breed appearances as their normal‐sized counterparts (Verhoef & Rijs, 2014). The F1 offspring produced by a crossing between bantam and normal‐sized chickens are expected intermediate in size, and offspring of F2 generation contain a range of segregating phenotypes (Wandelt & Wolters, 1998). Many neo‐bantam breeds were developed in the past decades mediated mainly by hobby breeders for different cultural reasons (Bortoluzzi et al., 2018; Dana et al., 2011), resulting in diverse historical and genetic backgrounds. Currently, almost every Dutch native breed has a corresponding neo‐bantam counterpart (Verhoef & Rijs, 2014).

However, our knowledge about bantam is very limited when it comes to the historical background and genetic mechanism. Understanding the bantam phenotype and the underlying genetic basis requires a wide variety of bantam chicken resources, as the genome landscape is influenced by various factors. The genetic background differs in Dutch chicken breeds (Bortoluzzi, Bosse, et al., 2020), and the backcrossing performed by breeders complicates the genetic tracing of bantam origin (Bortoluzzi et al., 2018), as well as the selective breeding for other phenotypes besides bantam, such as plumage color and comb morphology. Various bantams in the Dutch breeds contain a unique and documented history of crossbreeding, which enables us to disentangle the genetic nature of the bantam phenotype. In this study, we used whole‐genome sequence data for comprehensive genomic analyses to investigate the genetic architecture of the bantam phenotype. Understanding the genomic basis of the bantam phenotype will help to understand how the practice of bantam‐orientated crossbreeding has contributed to and reshaped the phenotypic and genetic heterogeneity of bantam chickens. The utility of evolutionary analytical approaches in this study provides a phylogenetic perspective of how the genetic variations were subjected to the convergent or divergent human‐mediated selection.

Given the complex history and limited knowledge of the bantam phenotype, the objectives of this research are as follows: (1) identify the bantam‐associated genes in Dutch chicken breeds; (2) investigate the characteristics of associated genes in historical heterogeneous groups of bantam; (3) understand the molecular mechanisms underlying the bantam phenotype.

## 2 MATERIALS AND METHODS

### 2.1 Sample collection and heterogeneous bantam backgrounds

Genomic DNA was isolated from 135 samples of 37 breeds (Table S1). We collected samples representing: (1) true bantams, which include the Dutch true bantam breeds and true bantams of Asian origin; (2) normal‐sized Dutch traditional native breeds; and (3) neo‐bantam counterparts of the Dutch native breeds. We collected information from breed associations on the specific bantam breeds that have been used to create the neo‐bantams. From the crossbreeding record, the Dutch breeds show various and divergent bantam origins among breeds (Table S2). The potentially nonuniform and heterogeneous genetic background in the population is suggested by the observation that some of the neo‐bantam breeds share the same ancestral bantam breeds. Based on the neo‐bantam crossbreeding record, we identify three groups derived from the three major bantam sources reported, which are the Dutch bantam (group 1), Sebright and Java bantam (group 2), and other miscellaneous bantams including south‐east Asian and productive breeds (group 3). In the three group‐based genome‐wide association studies (GWAS), we correspondingly analyzed the pairs of neo‐bantam breeds and normal‐sized native counterparts.

### 2.2 Whole‐genome sequencing and variant calling

Samples collected were sequenced on the Illumina HiSeq 3000 platform, including samples described in previous studies by Bortoluzzi, Bosse, et al. (2020) and Bortoluzzi, Megens, et al. (2020); in total, we achieved 135 samples of 37 breeds. The number of animals for each breed varies from 1 to 9. Whole‐genome sequence data were processed by using an in‐house analysis pipeline. In short, raw reads were first trimmed by Sickle (Joshi & Fass, 2011) using paired‐end mode. The cleaned reads were mapped to the latest Red Jungle fowl reference genome assembly, build GRCg6a (GenBank Accession: GCA_000002315.5) by using default options in BWA‐MEM (version 0.7.17) (Li & Durbin, 2009). With the markdup option in sambamba v0.6.3 (Tarasov et al., 2015), duplicated reads were removed. Mapping quality and coverage of the aligned samples were assessed with Qualimap v2.2 (Okonechnikov et al., 2016). Variant calling for SNPs and InDels was performed using Freebayes (Garrison & Marth, 2012) with the following criteria: minimum base quality 10; minimum mapping quality 20; and alternative calls need to have alternate fraction >0.2 and min‐alternate‐count >2.

### 2.3 Quality control

Variant quality control was performed using information from the depth of coverage, call rate, and minor allele frequency. We used VCFtools (Danecek et al., 2011) to include variants with a quality score over 10, call rate more than 0.8, a mean depth between 3 and 100, and genotypes with a depth between 3 and 100. When more than one alternative allele is present at one genomic position, in order to track all possible alternatives, multi‐allelic loci were split into few bi‐allelic sites by using BCFtools (Li et al., 2009) and PLINK (V1.9) (Chang et al., 2015). We assigned different names for the alleles to avoid ambiguities, which ensures the use of complete information for every locus. Rare variants were then filtered out by using a threshold of minor allele frequency <0.05.

### 2.4 Population structure analyses

The population structure of Dutch chickens was estimated by the principal component analysis (PCA) and neighbor‐joining (NJ) tree. We calculated the principal components using PLINK (V1.9) (Chang et al., 2015) with all genomic autosomal variants and visualized the first two principal components using ggplot2 (v3.1.0) (Wickham, 2016). The 1‐ibs distance matrix was computed in PLINK and then converted to a NJ tree using the R package “ape” (Paradis & Schliep, 2019). The phylogenetic tree was visualized by using FigTree v1.4.4 (http://tree.bio.ed.ac.uk/software/figtree/). Additionally, in order to estimate the relative genetic relationships within the grouped population, the phylogenetic tree analyses were conducted in a similar fashion within each group using only neo‐bantams of each group and the bantam sources.

### 2.5 Association study

Genome‐wide association studies were performed based on a grouping strategy. In this strategy, we identified three groups based on heterogeneous bantam backgrounds (Table S2), and each group contains matching pairs of normal‐sized breeds and corresponding bantam counterparts as the individual case and control phenotype. The number of individuals is 34 in group 1 (15 cases and 19 controls), 66 in group 2 (25 cases and 41 controls), and 35 in group 3 (12 cases and 23 controls). In each group, we employed the complete set of SNPs and InDels, and fitted a univariate linear mixed model including a relatedness matrix as the random effect by using the program GEMMA (Zhou & Stephens, 2012). The identity‐by‐state (IBS) relatedness matrix was computed by using the subset of SNPs corresponding to the chicken 60K SNP array, which is a good representation of Dutch chicken diversity (Bortoluzzi et al., 2018) and is computationally less demanding. The relatedness matrix was computed using (‐‐distance square ibs) in PLINK (V1.9) (Chang et al., 2015). The significant level *p*‐values were derived from the Wald test. The corresponding significance threshold followed the suggestive threshold of *p*‐value ≤ 5 × 10^−8^. A genomic control inflation factor lambda was calculated in each study to evaluate the confounding due to population stratification. The GWAS results were visualized using the R package qq‐man (Turner, 2018).

### 2.6 Structural variation analysis

In order to investigate whether structural variation (SV) affects the bantam phenotype, the association analyses with SVs were performed as described in the GWAS analyses. Considering the peak signals of the three groups, we detected structural variation (SV) on chromosomes 1 and 4 by using Smoove (https://github.com/brentp/smoove). Smoove combines various SV tools and removes spurious alignment reads to reduce the noise. Smoove first extracts discordant and split‐reads from the alignment BAM file per individual. Subsequently, Lumpy (Layer et al., 2014) is used to call SVs, and genotyping of SVs is performed using SVtyper (Chiang et al., 2015) after merging all SVs to generate a consensus output across the samples. Mosdepth (Pedersen & Quinlan, 2018) was used to further filter SV calls, to discard reads from regions where the sequencing depth of split or discordant reads was extreme (>1000), to remove regions that contribute to spurious calls. Duphold (Pedersen & Quinlan, 2019) was used to annotate depth changes within and on the breakpoints of SVs.

### 2.7 Meta‐analysis

A meta‐analysis was conducted to combine the evidence from independent GWAS analyses of the three groups by using a fixed‐effects inverse‐variance weighting approach. Because the three group‐based analyses were conducted in a uniform way, we used the classical estimate approach in METAL (Willer et al., 2010), in which each study provides the effect size of variants (beta), and the standard error was used to weight the study according to the inverse of squared standard error. In addition, we applied a genomic control correction for the *p*‐value. The between‐study heterogeneity was tested and addressed using ANALYZE HETEROGENEITY in METAL, which additionally evaluates the (in‐)consistency of the effect among studies. Additionally, to minimize the effect of population structure for comparison, we tested the overall association without using the grouping strategy. We performed the fifth association analysis directly pooling all 135 individuals together. The analysis of the “pooled” GWAS was conducted in the same fashion as described in group‐based GWAS. Finally, although the bantam is not considered as a sex‐linked phenotype according to its mode of inheritance, a sex‐linked analysis was performed in order to examine and confirm this. We first inferred the gender of individuals using sequence coverage on the Z and W chromosome. The ratio of sequence coverage (W/Z) was used to discriminate between ZZ (male) or ZW (female) individuals. The sex‐linked association analysis was performed in three groups, respectively, and the results were synthesized in the meta‐analysis. The gene associated with sex‐linked dwarfism (*GHR*) and the flanking region was investigated in more detail.

### 2.8 Annotation of the variants

The significant GWAS variants were further annotated for protein‐coding genes or known noncoding elements. In order to have eligible associations and reasonable control for the false positive, the significantly associated variants were selected using the conventional threshold of *p* ≤ 5 × 10^−8^. This roughly corresponds to a Bonferroni correction for 1 million common variants to maintain a 5% genome‐wide false‐positive rate (Panagiotou et al., 2012). We used Ensembl gene sets (version 95) for annotation including both coding and noncoding genes. We firstly annotated the variants based on their chromosomal position and distance to known genes. We reported significantly associated variants located within a gene range and the 1‐kb up‐ and downstream regions to consider the promoter region. Secondly, we used Variant Effect Predictor (McLaren et al., 2016) to further estimate the potential effect of variants (‐‐biotype ‐‐buffer_size 5000 ‐‐check_existing ‐‐distance 5000 ‐‐sift b ‐‐species gallus_gallus ‐‐symbol). We investigated and annotated the variants with existing SNPs, the affected gene, and the effect of the variant on the transcripts. To compare the results of the three association studies and the meta‐analysis, we visualized the shared and unique genes or variants among the four analyses using a Venn diagram (Heberle et al., 2015). Genes were listed when there is at least one variant annotated within the region. Functional annotation was performed using PANTHER v.11 (Mi et al., 2019) based on the chicken to human one‐to‐one orthologues. The Gene Ontology (GO) enrichment analysis was then conducted using the R package clusterProfiler (Yu et al., 2012).

### 2.9 Genetic differentiation (*F*_ST_) and haplotype sharing (IBD)

Haplotypes were phased for all individuals per chromosome using Beagle (version 5.0) (Browning & Browning, 2007) with a sliding window size of 0.02 and 0.01 cM overlap between adjacent windows. The analysis consisted of 12 iterations and the chromosomal genetic distances are based on (Elferink et al., 2010). Identity‐by‐descent (IBD) segments were detected by Refined‐ibd (Browning & Browning, 2013). Note that our goal was not to detect segments that are derived from a single co‐ancestor, but segments that come from the same population. Therefore, in order to detect these segments, we set relatively flexible criteria as follows: window = 0.06 cM, length = 0.03 cM, trim = 0.001 cM, finally LOD = 3.

To infer the relative fraction of haplotype shared with the traditional bantam versus the normal‐sized counterpart, we studied the relative IBD (rIBD) frequency in each group. We followed the rIBD calculation specified in Bosse et al. (2014). In short, the count of IBD segments (cIBD) that is shared between the traditional bantam and neo‐bantam was computed in windows of 10 kb and normalized for the total possible pair‐wise comparison (tIBD). The normalized IBD (nIBD) was then compared with the nIBD computed between normal‐sized counterpart and neo‐bantam. The relative value of nIBD sharing was defined as rIBD.

Count of IBD segments that were shared between the traditional bantam and neo‐bantam: cIBD_Bantam_Neo‐bantam_.

Total possible pair‐wise comparison between the traditional bantam and neo‐bantam: tIBD_Bantam_Neo‐bantam_.

Normalized IBD between neo‐bantam and traditional bantam: nIBD_Bantam_Neo‐bantam_ = cIBD_Bantam_Neo‐bantam_/tIBD_Bantam_Neo‐bantam_.

Normalized IBD between normal‐sized counterpart and neo‐bantam was computed respectively: nIBD_Counterpart_Neo‐bantam_ = cIBD_Counterpart_Neo‐bantam_/tIBD_Counterpart_Neo‐bantam_.

The relative value of nIBD sharing: rIBD = nIBD_Bantam_Neo‐bantam_ − nIBD_Counterpart_Neo‐bantam_.

If rIBD > 0, the neo‐bantam haplotypes within the region are considered more similar to the traditional bantam breeds compared to their normal‐sized counterparts, and vice versa.

To measure the genetic differences between groups, we estimated the fixation index (*F*_ST_) between the neo‐bantam with either their inferred bantam source or with the normal‐sized counterpart from the same group. The *F*_ST_ was calculated in windows of 10 kb using Vcftools with the method from Weir and Cockerham (1984). We performed a similar comparison as for rIBD, the *F*_ST_ between neo‐bantam and traditional bantam was compared against the *F*_ST_ between neo‐bantam and normal‐sized counterpart. The same approach was applied to the three bantam groups separately.

### 2.10 Haplotype analysis: “PhyloGWAS” for topology comparison between groups

To identify genetic regions that might have been selected from a common source, we borrowed the concept of “PhyloGWAS” (Pease et al., 2016) that correlates phenotypes with a subset of standing genetic variation regardless of overall relatedness. The “PhyloGWAS” approach was originally designed to detect the sorting of common ancestral variation among populations that are subject to common environmental conditions or similar selection pressures (e.g., parallel evolution). We applied a similar comparative strategy studying the different pairs of bantam and normal‐sized Dutch traditional breeds. In other words, the bantam variants selected from the common ancestry might group populations according to shared body size variation, despite the differences among breed pairs. To be more specific, such variants may be divergently fixed among the neo‐bantam and normal‐sized pairs, resulting in the expected topology that groups (neo‐)bantams together according to the phenotype rather than following the overall relatedness.

We constructed the genetic relatedness using regional information of candidate regions, both variant and haplotype based. For the significant variants identified in the meta‐GWAS, the genetic distance and phylogenetic tree were reconstructed similarly as described above including flanking regions. As for haplotype‐based analyses, sequences were extracted around the lead variants including 1 kb phased extending regions. As a next step, we computed 1‐ibs distance matrix and constructed the phylogenetic tree using the two‐phased haplotypes of every individual. In order to compare the haplotype blocks of each group and the linkage disequilibrium (LD) around the *HMGA2* gene, we computed the *r* ^2^ between the markers of this region against the highest associated variant.

## 3 RESULTS

### 3.1 Population structure of Dutch chickens

**FIGURE 1:**
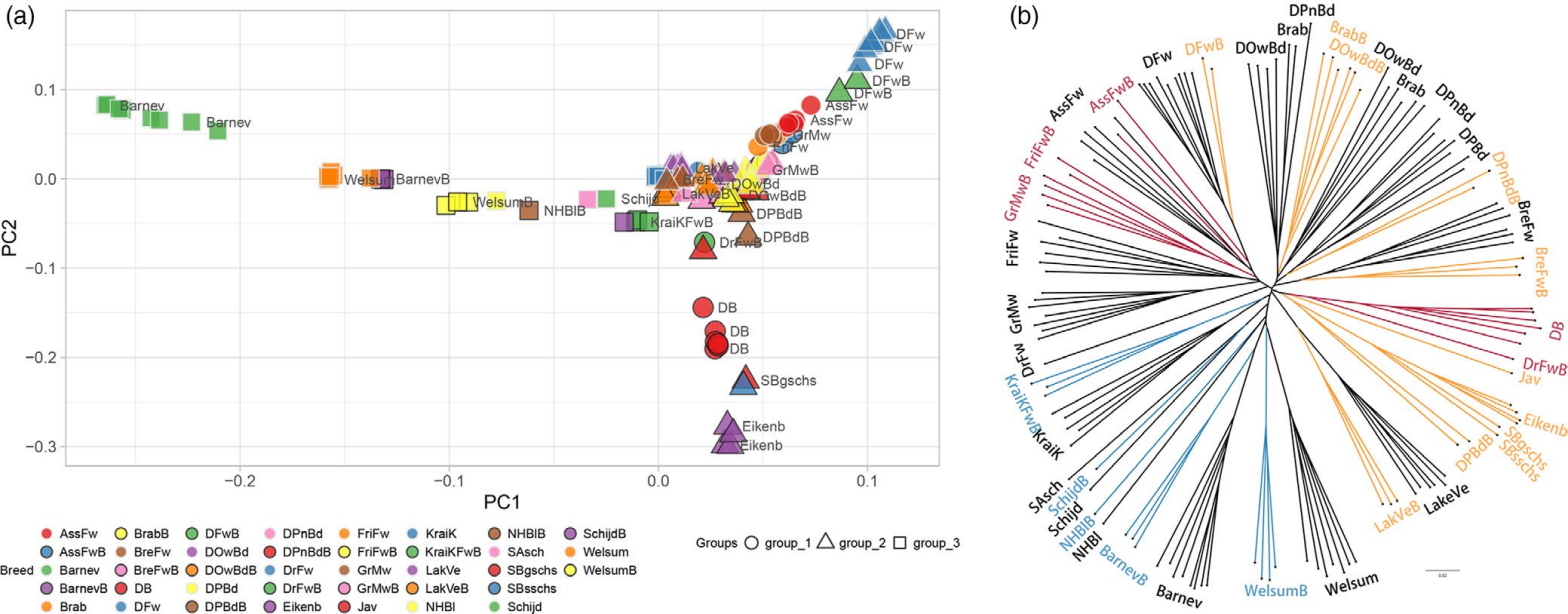
Population structure of Dutch chickens. (a) Principal component analysis, the breeds of chicken are displayed in different colors and shapes showing the three identified groups. (b) The unrooted neighbor‐joining tree of Dutch chicken presents 135 individuals, the bantam individuals have clades and nodes colored according to the phenotype and the groups: bantams in defined groups (group 1 in red, group 2 in yellow, and group 3 in blue), while the normal‐sized breeds are colored black. The abbreviation of the breed name is used according to Table S1

The Dutch chicken breeds show a diverse and admixed population structure. To perform a genomic analysis for the Dutch chicken population, whole‐genome sequence data of 135 individuals representing 37 breeds was generated. After filtering, 13.5 million autosomal bi‐allelic variants (including 11.6 million SNPs and 1.8 million InDels) were processed and used in the analyses to investigate the population structure. We classified the three groups according to the bantam sources, which are the Dutch bantam in group 1, Sebright and Java bantam in group 2, and other miscellaneous bantams such as productive breeds in group 3. A clear structure of the population (Figure 1a) is shown by the PCA and supported by the population tree (Figure 1b). Overall, neo‐bantam breeds show the distribution of closely clustering with the corresponding normal‐sized counterparts instead of a phenotype‐specific substructure, showing the higher genetic similarity between normal‐sized and neo‐bantam counterparts than among true bantams. Individuals from the same defined groups of bantam origin (Table S2) are generally grouped closely, especially for group 3. Some breeds in group 1 show considerable overlap with group 2, suggesting the overall normal‐sized breed relationship, whereas group 3 disperses from most of the population in the first component. Particularly, the within group relationships of neo‐bantams and true bantams are supported by additional population tree analyses (Figure S1C–E). For the second component, the true bantams, namely Dutch bantam (group 1), Sebright bantam, and Eikenburger bantam (group 2) separate from the majority of the traditional native breeds, which indicates definite divergence among breeds. Note that the grouping is based on the origin of bantam, not necessary on the relatedness of large fowls.

### 3.2 Group‐based GWAS results show different signals

**FIGURE 2:**
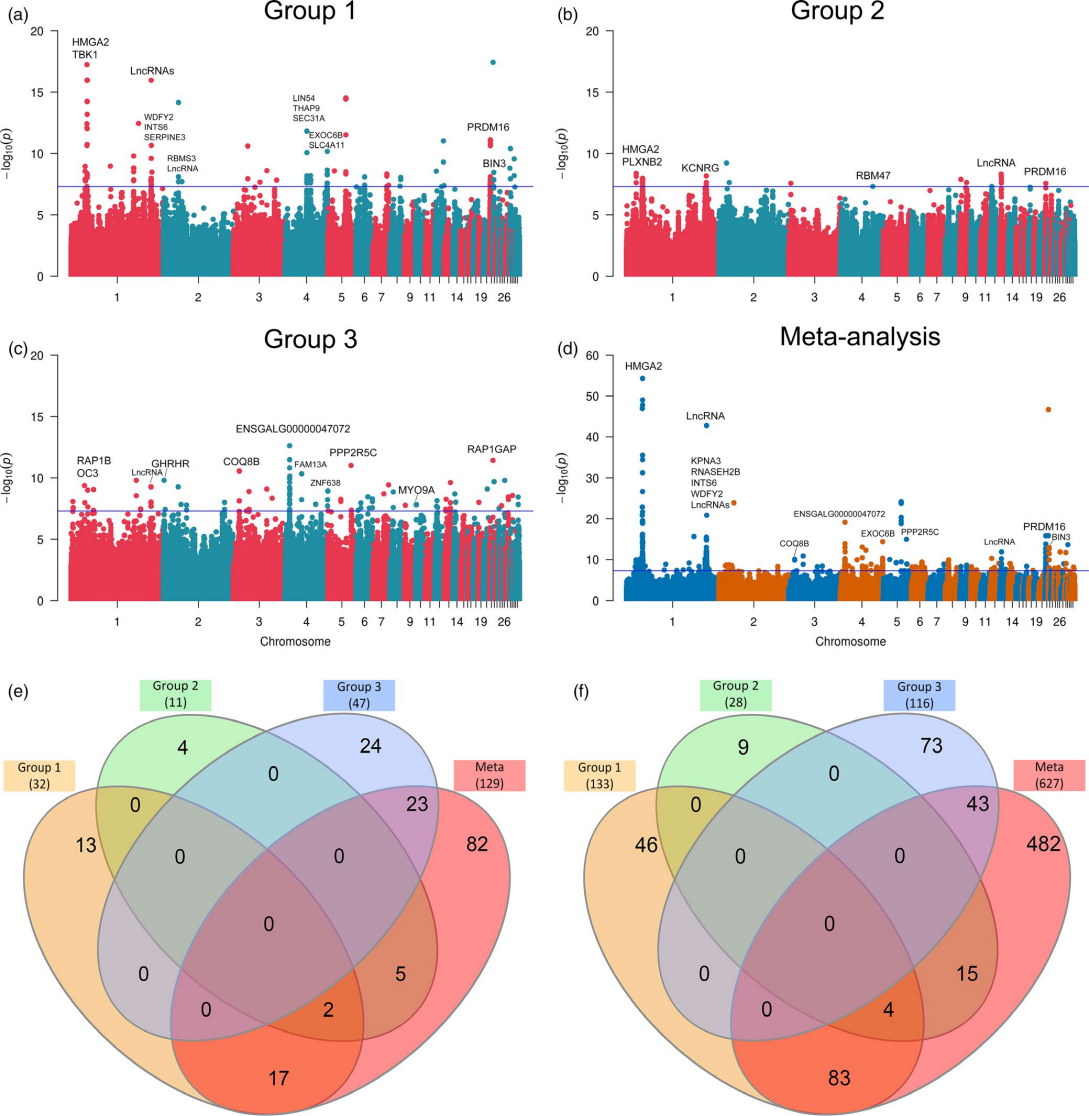
Manhattan plots show the genome‐wide association study results of (a–c) the three group‐based association and (d) the meta‐analysis. The −log_10_ *p* value on the *y*‐axis is plotted by autosomal variants on the *x*‐axis. The blue horizontal line indicates the suggestive cutoff threshold (*p* = 5 × 10^−8^). The variants are annotated with gene symbols, or long non‐coding RNAs (LncRNAs). Detailed gene information can be found in Table S4. The Venn diagrams display the comparison between groups 1 and 3 and the meta‐analysis, representing (e) associated genes and (f) significant variants among four association results

Because of the presupposed heterogeneous background of bantams, we performed GWAS on the bantam phenotype using autosomal genomic variants in the three bantam groups independently. Independent GWAS for each group was performed to analyze individuals with the bantam phenotype relative to the normal‐sized chickens within the corresponding group while accounting for the population structure. The lambda values of all GWAS were close to one (1.063–1.132), suggesting the population stratification was controlled. For the association studies with structural variants, we only found one putative bantam‐associated structural variant in group 2, whose effect however needs to be further studied (Figure S2). The analyses of the sex chromosomes confirmed that the bantam phenotype is not caused by a variant on sex chromosomes (Figure S3C,D). We did not find any association signal around the causative gene (*GHR*) for the sex‐linked dwarfism, which supports the inheritance mode of the bantam phenotype. The three GWAS results show autosomal variants statistically associated with bantam phenotypes; however, notably different signals are found among groups (Figure 2a–c). For group 1, we found a total of 133 variants surpassing the threshold (5 × 10^−8^), 78 of which are annotated with 32 known protein‐coding genes or noncoding elements. In group 2, in total 28 variants and 11 genes are significantly reported. With respect to group 3, 116 variants and 47 genes were denoted as significant. For the association, we found three major consistent signals in group 1 and group 2. First, we observe an interesting association on chromosome 1, located in the *High Mobility Group AT*‐*hook 2* gene (*HMGA2*). In this candidate gene, group‐specific signals reach the significance of *p* = 1.1 × 10^−16^ (in group 1) and *p* = 1.1 × 10^−8^ (in group 2). The significant variants are in the upstream and intronic regions of the gene. Other overlapping signals found in groups 1 and 2 are in the *PR*/*SET domain 16* gene (*PRDM16*) on chromosome 21, as well as a signal proximal 170–171 Mb on chromosome 1. However, the variants within these peaks show definite heterogeneity among the three groups.

When explicitly looking at each group, several unique associated genes are reported (Table S3). For instance, in group 2, we observed an associated SNP (NC_006089.5:g.16,453,888C>T) passes the Bonferroni significance threshold (*p* < 3.97 × 10^−9^) that is located in the intergenic region between the myosin IIIA (*MYO3A*) *and G protein*‐*coupled receptor 158* (*GPR158*) genes. We also identified a group 3 specific intronic variant (NC_006089.5:g.1,255,851T>C) in the *Growth Hormone Releasing Hormone Receptor* gene (*GHRHR*). Another example within group 3 is the highest associated SNP (rs13643124) on chromosome 4, located in the third intron of *ENSGALG00000047072*, which is orthologous to Bromodomain and WD repeat domain containing 3 (*BRWD3*). Neither this leading SNP nor the *BRWD3* gene was found as a candidate in the analyses of group 1 and group 2, suggesting distinct genetic determinants.

### 3.3 Meta‐analysis and comparison of associated variants and genes across studies

A meta‐analysis was performed across the three groups with the aim to detect shared loci and potentially new ones (Figure 2d). We used the meta‐analysis to combine the results of the three group‐based GWA studies and were able to detect shared bantam‐associated regions among different backgrounds. In total, the meta‐analysis resulted in 627 variants overlapping with 129 genes that surpassing the significant threshold (*p* ≤ 5 × 10^−8^), of which 15.9% have not been described by the Ensembl variation set (version 95). In total, 145 variants and 47 genes are shared by at least two of the four studies (three group‐based GWAS and the meta‐analysis). In the meta‐analysis, the most significant association (NC_006088.5:g.34,326,548G>C) is located within the first intron of *HMGA2* (*p* = 5.1 × 10^−55^), accompanied by other 52 variants enriched in this gene, suggesting the important role of *HMGA2* (Table S5). The direction of the allelic effect in the three studies is consistently positive, with the alternative allele (C) statistically associated with the bantam phenotype. The lead variant shows disparate frequency of the associated allele of 93.3%: 0% in bantam against normal‐sized in group 1, and in group 2 the frequency is 60.9%: 3.7%; whereas the frequency in group 3 is 27.8%: 0%. Particularly, no variant located in *HMGA2* is significantly associated with the bantam phenotype in group 3 (Figure S3B). The meta‐analysis also showed that an abundant number of associated variants (29 variants) were enriched for *PRDM16*. Interestingly, *HMGA2* and *PRDM16* are the only two genes commonly associated in group 1, group 2, and the meta‐analysis (Figure 2e). As for common variants between analyses, three significantly associated SNPs (rs313721485, rs313723493, and rs1058489589) are in the upstream and intronic region of *HMGA2*, while another SNP (rs735861847) is located in the first intron of *PRDM16* (Figure 2f). Although the aforementioned QTL (around 170–171 Mb on chromosome 1) has no specific SNP shared between the groups, the region stands out in the meta‐analysis too. The meta‐analysis revealed a candidate region peaks around 171.30 Mb (*p* = 2.1 × 10^−47^). The reported QTL covers several functional genes, for example, coding for WD Repeat and FYVE Domain Containing 2 (*WDFY2*), serpin family E member 3 (*SERPINE3*), integrator complex subunit 6 (*INTS6*), tripartite motif containing 13 (*TRIM13*), and noncoding elements, for example, *ENSGALG00000053256* and *ENSGALG00000052822*. By comparison, group 1 shows a significant association at 171.30–171.58 Mb, while group 2 and group 3 have candidates around 170.70 and 170.91 Mb, respectively. The subtler differences in the interval between three association studies resulted in a proximal associated region in meta‐analysis. The heterogeneity test in the meta‐analysis confirmed that 48.1% of the significant variants were heterogeneous. Typically, many variants reach significance in one analysis, while in other analyses they are either not associated or inconclusive. From the Venn diagram (Figure 2e,f), group 3 does not share any significant gene or variant with the other two groups, implying heterogeneous genetic backgrounds between the three bantam groups. In addition, the meta‐analysis also identified many unique variants and genes, showing the potential of discovering new genetic loci that might not reach significance in the single group GWAS. For example, four missense mutations were identified in the meta‐analysis, of which one (NC_006088.5:g.34,324,401C>A) is expected to alter the 2nd amino acid residue from serine to arginine in the aforementioned candidate gene *HMGA2*.

Furthermore, we compared the results of the meta‐analysis and a “pooled” GWAS without using the grouping strategy (Figure S3A); the latter is directly pooling all cases and controls in one analysis. With the same sample size, the meta‐analysis shows higher significance levels than the “pooled” GWAS without the grouping strategy. Moreover, although the major associations were also revealed by the “pooled” GWAS as in the meta‐analysis, the majority of the signals failed to be detected using this approach. This suggests that the variants are likely only associated with a subset of the population, and the tests may lose power by simply pooling animals together. These results demonstrate the power of meta‐analysis in detecting the potential association between analyses.

With respect to the functional annotation of significant genes across studies (170 genes), we identified GO terms related to development, though the enrichment analysis was not significant (Figure S4, Table S6), some of the GO terms are of explicit interest: bone morphogenesis, regulation of skeletal muscle tissue development, bone development, and negative regulation of cellular response to growth factor stimulus.

### 3.4 Exploring HMGA2 associated region: *F*_ST_ and rIBD analyses

**FIGURE 3:**
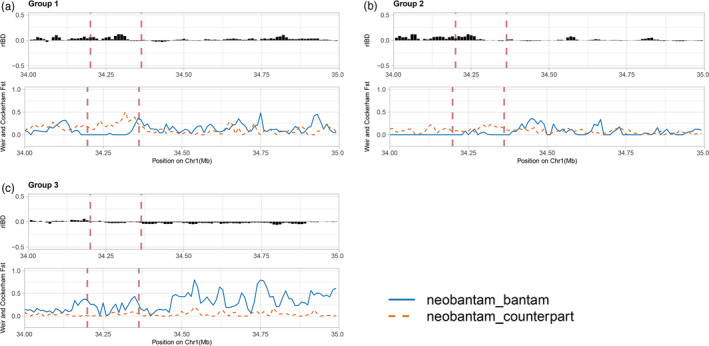
Distribution of the rIBD and *F*_ST_ of the three groups (a–c) around the HMGA2 corresponding interval (34–35 Mb). The *x*‐axis displays the chromosomal coordinates, while the *y*‐axis shows the value of rIBD and *F*_ST_. In each group, the upper panel shows the estimation of rIBD. The positive value of rIBD suggests more similarity between neo‐bantam and bantam source than between neo‐bantam and normal‐sized counterpart; the negative rIBD value, on the contrary, shows the high haplotype sharing between neo‐bantam and normal‐sized counterpart. The lower panel displays the *F*_ST_ estimation, the blue solid line represents the *F*_ST_ between neo‐bantam and bantam source, while the orange dashed line displays *F*_ST_ between neo‐bantam and normal‐sized counterpart

To further investigate the bantamization history and the introgression pattern of neo‐bantam breeds, we explored the haplotype sharing and genetic differences on chromosome 1 using *F*_ST_ and relative IBD sharing (rIBD). We computed the *F*_ST_ and shared IBD segments between neo‐bantams and their normal‐sized counterpart, as well as that between neo‐bantams and the corresponding bantam source. Overall, we observed a generally lower mean *F*_ST_ (0.03–0.07) between the neo‐bantams and their normal‐sized counterparts than between the neo‐bantams and the bantam sources (0.04–0.22). Similarly, we found more extensive and longer shared IBD segments between neo‐bantams and normal‐sized counterparts than between neo‐bantams and bantam sources (Figure S5), in line with the expected close genetic relatedness between the native breeds and neo‐bantams. However, when zooming in into the bantam‐associated interval containing *HMGA2* on chromosome 1, positive rIBD signals were found in groups 1 and 2, which suggests that the neo‐bantams share more similar haplotypes with bantam sources in this interval than with normal‐sized counterparts (Figure 3). The regional introgression from the bantam sources to neo‐bantams is also supported by a lower regional *F*_ST_ estimation. As a contrast, the regional negative rIBD and higher *F*_ST_ displayed in group 3 are in accordance with the absence of an association signal. Another example is the regional pattern of *F*_ST_ and rIBD in the group 3 specific interval on chromosome 4 (Figure S6). Our results confirm the introgression of these regions in which the neo‐bantams are more similar to their bantam sources, suggesting a regional strong introgression from the bantam source rather than from the normal‐sized counterpart.

### 3.5 Constructing haplotype “PhyloGWAS” across breeds

**FIGURE 4:**
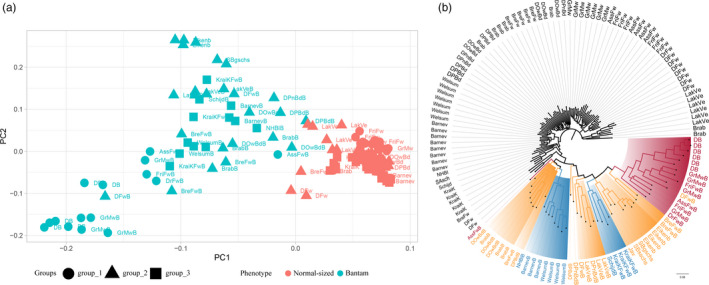
Principal component analysis and neighbor‐joining tree of Dutch chickens using all the significant markers. (a) The phenotype of individuals is displayed by colors, and groups of individuals are shown by different shapes. (b) All individuals are displayed by clades and nodes with different colors (group 1 in red, group 2 in yellow, and group 3 in blue), the normal‐sized breeds are colored black. The bantam individuals are highlighted with background colors by the three groups

Finally, to investigate bantam introgressed haplotypes that might have been selected from segregating ancestral variation, we estimated the genetic relationship among groups using both information from overall significant loci and haplotype blocks within intervals. Specifically, the PCA and phylogenetic tree were used to test if the topologies of genetic variants came from confounding effects. Firstly, we analyzed this using all the significant variants (*n* = 755) across the genome. A clear separation between bantams and normal‐sized individuals is observed in both the PCA and the phylogenetic tree (Figure 4). Interestingly, the associated variants not only separated the case and control individuals but also distinguished the bantams of group 1 from the rest of the bantams. More variations were found within the (neo‐) bantams than in the normal‐sized breeds, supported by the longer branch lengths in the phylogenetic tree and the disperse distribution of bantam breeds in the PCA. Moreover, the topology of all the bantam breeds from group 1 demonstrates a stronger genetic relatedness than the other two groups. These results show that although we observed limited overlap in associated genetic variants between three groups, there is still a clear overall similar selection for the bantam phenotype in the neo‐bantam breeds. Secondly, we narrowed down to the haplotypes of specific lead variants. The associated haplotype in each group confirmed that the three groups have clearly different association patterns as well as haplotype blocks (Figure S7A–C). In particular, the haplotypes in group 1 and group 2 both showed association patterns in the *HMGA2* related region whereas the haplotypes of group 3 showed no association in the corresponding region. Moreover, the regional phylogenies illustrate the presence of multiple haplotypes in groups. For example, the haplotype around the lead SNP (NC_006088.5:g.34,326,548G>C) comprised in *HMGA2* (Figure S8A) displays a general cluster of haplotypes stemming from bantams from group 1, with a few other bantam breeds from group 2 and even fewer from group 3. As this SNP is located in a clear introgressed region, the surrounding haplotype is almost completely associated with the bantam phenotype in group 1, while the level of association gradually declines in groups 2 and 3. The phylogenetic topology in group 1 demonstrated that the bantams were grouped in close clades, but we also observed the haplotypes of few bantam individuals (e.g., Dutch Bantam) were grouped next to this lineage showing the haplotype diversity. This implies that even when studying a small haplotype block (2 kb), the strongly associated SNP in different groups may be present on multiple haplotypes. The haplotype diversity can be further evidenced by the illustration of LD measured in the three groups. In agreement with the haplotype patterns, the haplotype blocks in group 2 show lower LD but are relatively longer in size when compared with the haplotype blocks of group 1 (Figure S7D). The approximate intervals with high LD (*r* ^2^ > 0.9) in groups 1 and 2 are between 34.32–34.33 Mb and 34.32–34.36 Mb, respectively.

This selection on multiple bantam haplotypes across groups and breeds, considered together with the phenotype‐related separation shown by overall associated variants, could potentially be explained by the convergent selection on the ancestral variations. The comprehensive allelic heterogeneity is confirmed by similar distinct patterns of other associated variants (Figure S8).

## 4 DISCUSSION

### 4.1 Bantam breeding information enables untangling the complexity of the bantam phenotype

The human‐mediated bantamization reshaped the genetic characteristics of neo‐bantam chickens through crossbreeding. Here, we focus especially on the historical bantam breeding in the Dutch indigenous breeds. Bantam breeding in the Netherlands has been performed by hobby breeders adhering to breed standards. Despite the absence of an exhaustive crossbreeding scheme, the complexity of the bantam phenotype was revealed by our investigation showing that different (sub‐)types of bantam breeds were utilized.

Body size, known as a highly polygenic trait in animals, has distinct forms of the phenotype presented in different breeds. The success of using GWAS to identify genetic variants in complex traits and diseases has been remarkable (Hardy & Singleton, 2009; Schaid et al., 2018). An appropriate GWAS population is based upon the matching of cases and controls and avoids the cryptic relatedness that might lead to population stratification (Tam et al., 2019). Here, we utilized the unique model of Dutch traditional chicken breeds involving matching counterparts of the normal‐sized native breed and neo‐bantam breed. Previous studies on dwarfism have focused on specific genetic variants in the genome (Agarwal et al., 1994; Boegheim et al., 2017; Burnside et al., 1992; Wu et al., 2018), whereas diverse backgrounds and heterogeneous phenotypes in different breeds have received little attention. Additionally, the formation of neo‐bantam breeds requires us to account for the historical records of bantam crossbreeding and to study the bantam phenotype within specific groups.

In this study, we integrated within bantam group association studies and a meta‐analysis across the populations to study the unknown genetics underlying the bantam phenotype. Such an approach has successfully identified genetic loci in previous studies, such as human height (Weedon et al., 2008), bovine stature (Bouwman et al., 2018), and canine hypothyroidism (Bianchi et al., 2015). The grouping in our study is explicitly based on the source of bantam used and shared during crossbreeding, and not necessarily on the genetic relatedness of the normal‐sized breeds, which is nevertheless largely consistent with previous studies (Bortoluzzi et al., 2018; Elferink et al., 2012). We focused on the bantam genetic variants shared across various breeds with similar historical bantamization background and used the matching pairs of cases and controls (the normal‐sized and its neo‐bantam breed), which are breeds with the same appearance but only differ in size. This allowed us to use a relatively small population to detect eligible bantam‐associated genes. We also obtained many more significant signals in the meta‐analysis at detecting bantam‐associated variants, outcompeting pooling the breeds while treating bantams as one single population. With breed pooling, cryptic population structure in the genetic background was introduced to the study, therefore variants that should show association in a subset of the population failed to be discovered.

### 4.2 Heterogeneity of bantam and its sources

Our study provides insight into the heterogeneity of genotype–phenotype associations across diverse bantam ancestral groups. Genetic heterogeneity was observed for the lack of sharing significant variants in the four major association analyses (three group‐based GWAS and the meta‐analysis), especially for group 3. The haplotype blocks surrounding *HMGA2* reported in group 1 and group 2 are significantly associated with bantam phenotype, but this association is not observed in group 3. Although there is an indication that the lead SNP is not carried by any normal‐sized individuals in group 3, the frequency of the bantam‐associated alleles and haplotype is too low to reach a statistical significance. Based on the size of LD blocks observed in groups 1 and 2, we anticipate that multiple haplotypes are associated comprising the causal variant. Furthermore, the definite heterogeneous genetic architecture was illustrated in both the variant heterogeneity test and regional phylogeny structure, showing the complexity of the bantam trait is composed of distinct subtypes.

There are three basic sources that can introduce heterogeneity in our study. First, heterogeneity can be caused by a nonstandard phenotypic definition. In the case of complex traits, phenotypic determination sometimes is difficult to standardize and define, almost inevitably resulting in phenotypic heterogeneity. Previous studies suggest that dwarfism in chicken is a phenotype involving many forms of variation (Agarwal et al., 1994; Andersson & Georges, 2004; Boegheim et al., 2017; Wu et al., 2018). In agreement with this, we observed differences in the reduction of standard body weight across breeds (Figure S1A,B). Second, the heterogeneity may result from different ancestral origins (Evangelou & Ioannidis, 2013). In our study, we utilized the bantam historical record as a key basis for the association study. The cryptic ancestral groups are interpreted from the crossbreeding histories and confirmed by the definite differences in association patterns between the three groups. This is further supported by the introgression signals showing rIBD and *F*_ST_ fractions across the three groups are different. Lastly, we observed heterogeneity in the allele effect across different analyses. We performed a heterogeneity test in the meta‐analysis to estimate the variance effect between studies (Table S4). As described above, we confirmed that multiple variants are associated with only a subset of the population, and the effect of alleles can be found different across groups. Furthermore, the haplotype analysis demonstrates that one specific haplotype could not completely explain the phenotypic differences among groups. Within one group, even within one breed, the variation in haplotypes cannot present a complete phenotype‐specific topology, which is evidence of genetic heterogeneity and haplotype diversity. The haplotype diversity is additionally evidenced by the LD between markers within the associated region (Figure S7D). In groups 1 and 2, the relatively short size of haplotype blocks and the degree of LD observed in the nearby sites suggested that the genetic variants surrounding the lead variant are not completely fixed in the bantam breeds. This implies the presence of more than one haplotype where the most significant SNPs are located on. The genomic analyses in this study show the fact that the Dutch chicken breeds contain diverse and complex bantam resources. In future studies, in order to control the unknown phenotypic and genetic heterogeneity, a standard phenotypic definition and measurement are desirable, especially for meta‐analyses (Evangelou & Ioannidis, 2013).

### 4.3 Overview of candidate genes found in GWAS

Bantam‐associated genes were investigated for function in the growth of animals. Our results show that bantam is a typical polygenic trait, thus the integrative approach we implemented provides a unique opportunity to study it. The overlap between the meta‐analysis and group‐based GWAS demonstrates two genes in particular: *HMGA2* and *PRDM16*. Both of these genes have been reported to correlate with the growth of myoblasts (Li et al., 2012; Seale et al., 2008). Variants (i.e., deletion, missense, and UTR variants) in *HMGA2* have been reported to be associated with reduced growth and stature in several other species, including human, mouse, rabbit, dog, horse, and cattle (Bouwman et al., 2018; Carneiro et al., 2017; Frischknecht et al., 2015; Rimbault et al., 2013; Weedon et al., 2007; Zhou et al., 1995). *HMGA2* controls the proliferation of myoblasts and muscle development by regulating the expression of IGF2‐binding protein 2 (*IGF2BP2*), which subsequently regulates multiple genes important for cell growth (Li et al., 2012). A variant in *HMGA2* was previously reported to be correlated with body weight in chicken (Song et al., 2011), but our study is the first to report the association between *HMGA2* and chicken dwarfism. In our study, most of the variants in *HMGA2* are intronic and upstream variants and not directly affecting the protein‐coding sequence. The significant variants are in a region that spans from the upstream till the first intron of *HMGA2*, containing signals of introgression and selection from the bantam origin to the neo‐bantam, particularly in group 1. Similarly, variations detected in *PRDM16* were reported to be associated with growth and fatness traits in chicken and other species (Han et al., 2012; Seale et al., 2008; Wang et al., 2010). It is known that variation in *PRDM16* controls the cell fate switch between skeletal myoblasts and brown fat cells (Seale et al., 2008). Apart from that, the candidate region around 170–171 Mb on chromosome 1 stretches across a widely reported proximal 1.5 Mb QTL (170.52–172.04 Mb, Xie et al., 2012). The QTL region influences growth and body weight and contains lncRNAs and microRNAs (e.g., gga‐mir‐16‐1) that are reported to regulate the expression of growth‐related genes (Sheng et al., 2013; Wahlberg et al., 2009; Xie et al., 2012). In this candidate region, groups show slightly different refined intervals for the association, and candidate SNPs in each group do not overlap. In addition, the predicted consequences of the variants in this QTL are mostly intronic, upstream and downstream variations, which make it difficult to directly conclude the consequence on the expression of genes. Our study showed the importance of this QTL in regulating body growth in bantam chickens, the biological function and the genetic heterogeneity around this QLT should be confirmed and investigated in future studies. We found group 3 to extensively exhibit a heterogeneous association pattern compared to the analyses of groups 1 and 2. Specially, a unique signal on chromosome 4 peaks within the gene ENSGALG00000047072, a fruit fly *BRWD3* homologue. In humans, this gene correlates with intellectual disability and macrocephaly and may alter developmental signaling (Chen et al., 2015; Field et al., 2007).

### 4.4 Overall convergent selection and regional introgression

By comparison, the “PhyloGWAS” and haplotype analyses show clear haplotype diversity across the three groups, yet a potential convergent selection on the overall associated variants. For example, according to the presumed history of group 1, Dutch Bantam was repeatedly used as the (in‐)direct source of bantam donor, which can be supported by a relatively consensus haplotype around the lead variant shared between the bantams within this group (Figure S8A,B). In Dutch traditional chickens, it is likely that selection for the bantam phenotype was performed in multiple groups containing different underlying haplotypes with bantam alleles. As a result, the multiple haplotypes in different groups and breeds can undergo a convergent selection for the bantam phenotype in the Dutch populations.

Generally, due to the intense crossbreeding and selection for a similar appearance as their normal‐sized counterparts, the neo‐bantams are genetically closely related to their normal‐sized counterparts. Therefore, different from the majority of the genome, the regional high rIBD and low *F*_ST_ signals in neo‐bantams when compared to the bantam source indicate that these bantam‐related genomic regions undergo a stronger introgression from the bantam source rather than from the normal‐sized counterpart. Moreover, this suggests that most of the genomic contribution of the true bantam breeds is flushed out so that only haplotypes relevant for the bantam phenotype remain in the neo‐bantams.

Finally, the intronic and upstream variants found in the candidate list remain to be prioritized regarding their functionality. Currently, to improve the annotation of the causative alleles and functional regulatory elements, investigations are on the way performed by the international consortium Functional Annotation of Animal Genomes (FAANG) (Andersson et al., 2015), which will refine the causative variants and biological mechanisms that underlie the bantam phenotype.

Taken all together, we conclude that different bantam‐associated genomic regions are observed in the three groups of Dutch chicken breeds, accompanying by heterogeneity and diverse crossbreeding histories. Within the Dutch chicken populations, neo‐bantam breeds were derived from the normal‐sized counterpart by using bantams as the donor for the dwarf phenotype. Therefore, making use of matching pairs of (neo‐)bantam and normal‐sized breeds provided us with a powerful proxy to understand the bantam phenotype. We used genomic analyses to show that the bantam phenotype is a complex trait caused by multiple underlying genes, which shows heterogeneity across the historical groups. We report bantam‐associated genes in these group‐based studies, including *HMGA2* and *PRDM16*, some of them are reported to correlate with dwarfism in chicken for the first time. Among Dutch bantam breeds, we show the selection for the bantam phenotype is likely subjected to a convergent direction across populations. As a result of crossbreeding, neo‐bantams show regional introgression signals from the traditional bantam sources in the associated genomic regions. Overall, the genomic analyses on Dutch bantam breeds and the bantamization history demonstrate how human‐mediated crossbreeding diversely reshape the genome and phenotype.

## CONFLICT OF INTEREST

All authors declare no potential conflict of interest.

## Supporting information

Appendix S1

Fig S1‐S8

Table S1‐S6

## Data Availability

Whole‐genome sequence data were deposited in the European Nucleotide Archive (ENA). The data can be accessed through the three project numbers: (1) PRJEB34245, which described 88 traditional chickens from the Netherlands in a previous study (Bortoluzzi, Bosse, et al., 2020). (2) PRJEB39725, the data of 44 Dutch chickens has been submitted. (3) PRJEB36674, three samples (two Sebright bantams and one Sumatra) were described in a previously published study (Bortoluzzi, Megens, et al., 2020).
