# Supplementary material for: Heterogeneity of a dwarf phenotype in Dutch traditional chicken breeds revealed by genomic analyses
Source: Evol Appl. 2021 Jan 19;14(4):1095–108. doi: 10.1111/eva.13183 (PMC8061282; doi:10.1111/eva.13183)
Supplement: Supplementary file 1 — Appendix S1 [file EVA-14-1095-s002.pdf]

The description of Dutch chicken breed, the corresponding standard boody weight (in gram), and the calculated mean reduced ratio of body weight. For more characteristics of breeds, please refer to www.fao.org/dad-is .

**Breed Abbr.**	**Breed(in Dutch)**	**Size**	**Hen weight(g)**	**Rooster weight(g)**	**Mean reduced ratio**	**Hen ringsize(mm)**	**Rooster ringsize (mm)**	**Info**
AssFw	Assendelfter	Normal	1300	1700		15	13	https://dier-en-natuur.infonu.nl/huisdieren/185933-kippenras-assendelfter.html#karakter
AssFwB	Assendelfter kriel	Bantam	550	650	59.73%	10	11	https://www.kippenpagina.nl/kippenrassen/assendelfterkrielen.html
Barnev	Barnevelder	Normal	2750	3500		18	20	https://dier-en-natuur.infonu.nl/dieren/185918-kippenras-barnevelder.html
BarnevB	barnevelderkriel	Bantam	900-1000	1100-1200	66.30%	13	15	https://dier-en-natuur.infonu.nl/dieren/185945-kippenras-barnevelderkriel.html
Brab	Brabanter	Normal	2000	2500		16	19	https://dier-en-natuur.infonu.nl/dieren/185925-kippenras-brabanter.html
BrabB	Brabanterkriel	Bantam	750	1000	61.25%	NA	NA	https://dier-en-natuur.infonu.nl/dieren/185943-kippenras-brabanterkriel.html
BreFw	Kraaikop	Normal	1750-2250	2500-3000		NA	NA	https://dier-en-natuur.infonu.nl/dieren/185930-kippenras-kraaikop.html
BreFwB	Kraaikopkriel	Bantam	800-900	900-1000	61.48%	NA	NA	https://dier-en-natuur.infonu.nl/dieren/185941-kippenras-kraaikopkriel.html
DB	Hollandse kriel	Bantam	400-450	500-550		NA	NA	http://www.kurkiminiaturki.eu/holkriel.html
DFw	Hollands hoen	Normal	1800	2500		NA	NA	https://dier-en-natuur.infonu.nl/dieren/185924-kippenras-hollands-hoen-of-hamburgh.html
DFwB	Hollandse hoenkriel	Bantam	700-800	800-900	62.17%	NA	NA	https://dier-en-natuur.infonu.nl/dieren/185934-kippenras-hollandse-hoenkriel-of-hamburgh-kriel.html
DOwBd	Nederlandse uilebaard	Normal	1500-2000	1900-2500		16	18	https://dier-en-natuur.infonu.nl/huisdieren/185927-kippenras-nederlandse-uilebaard.html
DOwBdB	Nederlandse uilebaardkriel	Bantam	700	800	61.82%	11	13	http://www.kippenpagina.nl/kippenrassen/nederlandseuilebaardkrielen.html
DPBd	Nederlandse baardkuifhoenders	Normal	1500-1700	2000-2300		16	18	http://www.kuifhoenderclub.nl/site/
DPBdB	Nederlandse baardkuifhoenkriele	Bantam	700-800	900-1000	54.47%	12	13	http://www.kuifhoenderclub.nl/site/
DPnBd	Hollandse kuifhoenders	Normal	1400-1600	1900-2200		15	16	http://www.kuifhoenderclub.nl/site/
DPnBdB	Hollandse kuifhoenkrielen	Bantam	700-800	800-900	54.27%	11	13	http://www.kuifhoenderclub.nl/site/
DrFw	Drents hoen	Normal	1500	1900		13	15	https://dier-en-natuur.infonu.nl/dieren/185923-kippenras-drents-hoen-en-drents-bolstaarthoen.html
DrFwB	Drentse kriel	Bantam	600-700	700-800	58.60%	11	13	https://dier-en-natuur.infonu.nl/dieren/185932-kippenras-drentse-kriel-en-drentse-bolstaartkriel.html;https://www.kippenpagina.nl/kippenrassen/drentshoenkrielen.html
Eikenb	Eikenburger kriel	Bantam	550	650		11	12	https://www.kippenpagina.nl/kippenrassen/eikenburgerkriel.html
FriFw	Fries hoen	Normal	1400	1600		NA	NA	https://dier-en-natuur.infonu.nl/dieren/185922-kippenras-fries-hoen.html
FriFwB	Friese hoenkriel	Bantam	600-700	700-800	53.35%	NA	NA	https://dier-en-natuur.infonu.nl/dieren/185939-kippenras-friese-kriel-of-friese-hoenkriel.html
GrMw	Groninger meeuw	Normal	1600-1800	1800-2100		NA	NA	https://zeldzamerassen.nl/groningermeeuwenclub/beschrijving/
GrMwB	Groninger meeuw kriel	Bantam	700	800	58.90%	NA	NA	https://zeldzamerassen.nl/groningermeeuwenclub/beschrijving/
Jav	Java bantam	Bantam	500-550	600-675		NA	NA	https://dier-en-natuur.infonu.nl/dieren/192071-kippenras-javakriel.html
KraiK	Twents hoen	Normal	2000-2500	3000		16	20	https://dier-en-natuur.infonu.nl/dieren/185947-kippenras-twents-hoen.html
KraiKFwB	Twents kriel	Bantam	750-900	850-1100	65.42%	11	13	https://dier-en-natuur.infonu.nl/huisdieren/185940-kippenras-twents-kriel.html
LakVe	Lakenvelder	Normal	1600	2000		NA	NA	https://dier-en-natuur.infonu.nl/dieren/185944-kippenras-lakenvelder.html;https://nl.wikipedia.org/wiki/Lakenvelder_(kip)
LakVeB	Lakenvelderkriel	Bantam	700	800	58.13%	NA	NA	https://dier-en-natuur.infonu.nl/dieren/185938-kippenras-lakenvelderkriel.html
NHBl	Noord-Hollands hoen	Normal	3000-3500	3500-4000		NA	NA	https://dier-en-natuur.infonu.nl/dieren/185937-kippenras-noord-hollandse-blauwe-of-noord-hollands-hoen.html

NHBlB	Noord-Hollandse kriel	Bantam	850-950	1000	72.82%	NA	NA	https://dier-en-natuur.infonu.nl/dieren/185928-kippenras-noord-hollandse-kriel.html
Sasch	Sumatra	Normal	2250	2500		NA	NA	https://dier-en-natuur.infonu.nl/dieren/188463-kippenras-sumatra-het-zwarte-vechthoen.html
SBgschs/SBsschs	Sebright	Bantam	525-575	627-675		NA	NA	https://dier-en-natuur.infonu.nl/huisdieren/192911-kippenras-sebright.html
Schijd	Schijndelaar	Normal	1500-2000	2000-2500		17	19	https://www.kippenpagina.nl/kippenrassen/schijndelaar.html | http://www.oasll.com/schijndelaar.html
SchijdB	Schijndelaar kriel	Bantam	NA	NA		NA	NA	A bantam version is also available for this breed, but it hasn´t been standardized completely yet.
Welsum	Welsumer	Normal	2000-2500	2750-3250		18	20	https://www.kippenpagina.nl/kippenrassen/welsumer.html| https://dier-en-natuur.infonu.nl/dieren/185946-kippenras-welsumer.html
WelsumB	Welsumerkriel	Bantam	900-1000	1000-1200	60.56%	13	15	https://dier-en-natuur.infonu.nl/huisdieren/192896-kippenras-welsumerkriel.html
