# Supplementary material for: Heterogeneity of a dwarf phenotype in Dutch traditional chicken breeds revealed by genomic analyses
Source: Evol Appl. 2021 Jan 19;14(4):1095–108. doi: 10.1111/eva.13183 (PMC8061282; doi:10.1111/eva.13183)
Supplement: Supplementary file 2 — Fig S1‐S8 [file EVA-14-1095-s001.pdf]

Supplementary information

Figure S1 The breed reduced body weight and the phylogenic structure of the three groups.
Figure S2 The association studies of structural variations for three groups on GGA1 and GGA4 respectively。
Figure S3 Manhattan plot of GWAS results of the “pooled” analysis, HMGA2 analysis in group 3, and sex-linked analysis.
Figure S4 The enrichment analysis of Gene Ontology (GO) terms.
Figure S5 The histogram shows the comparison of the count of IBD fragments in the three groups.
Figure S6 Distribution of rIBD and Fst estimation on GGA4 the group 3 specific region.
Figure S7 The haplotype of genomic associated sites in the three groups.
Figure S8 NJ-tree topology of haplotype and PCA structure (page 9-10).

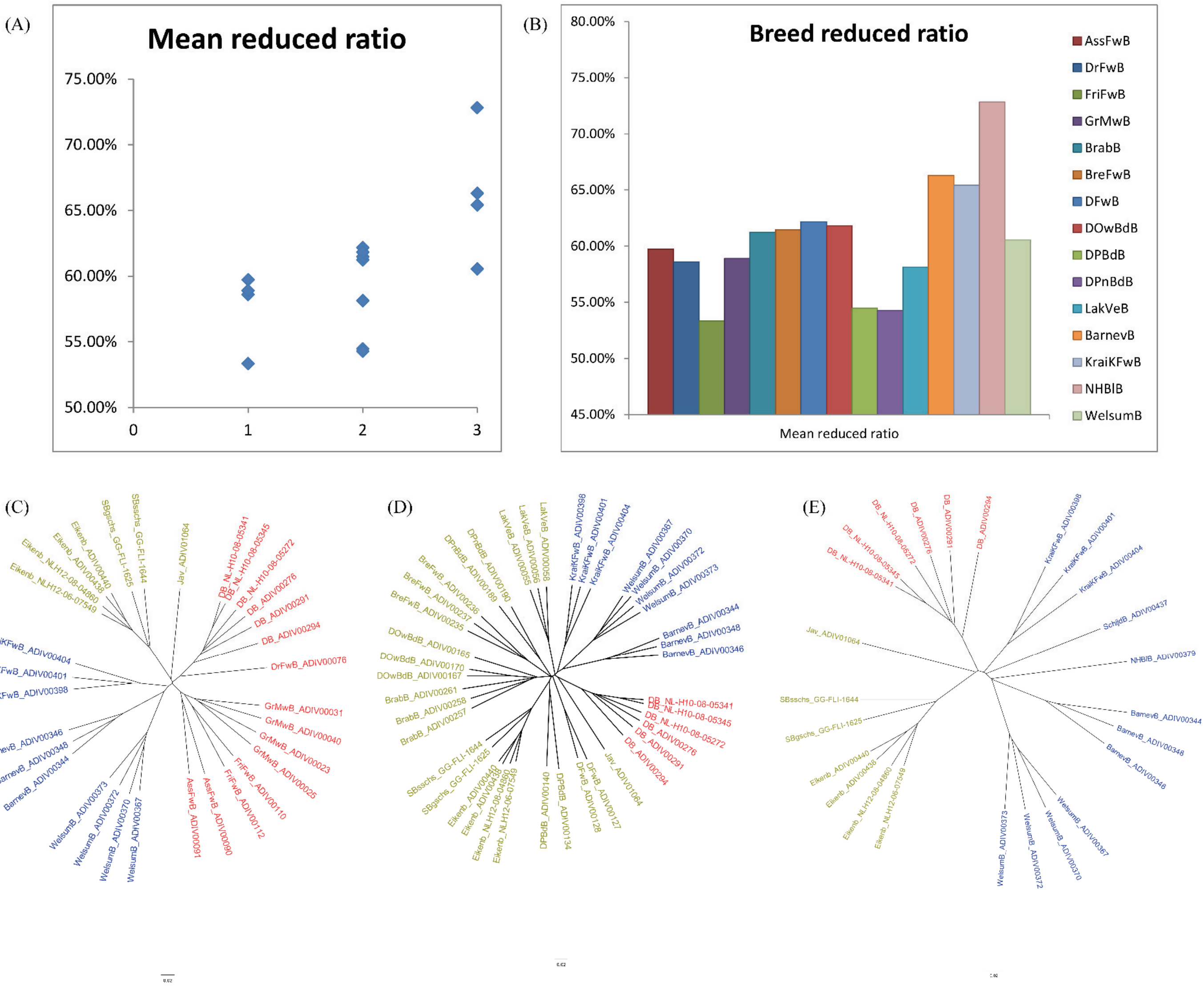

Figure S1 The breed reduced body weight and the phylogenic structure of the three groups. The Breed standard bodyweight of Dutch chicken is collected (Supplementary file 1) and the breed reduced body weight of each breed was summarized in three groups. Figure (A) shows the mean ratio of reduced body weight (shown in the y-axis) summarized according to three groups (displayed by the x-axis). Figure (B) shows the reduced ratio of breeds. The figures (C-D) show the NJ-tree topology of neo-bantams of each group and the bantams source in the complete dataset, namely Dutch bantam (group 1), Java bantam and Sebright bantam (group 2); true bantams in group 3 were not sampled in this study, therefore three representative neo-bantams that possess diversification are used to represent the group. The color scheme is based on the group, red nodes show individuals from group 1, yellow shows group2, and group 3 is in blue nodes.

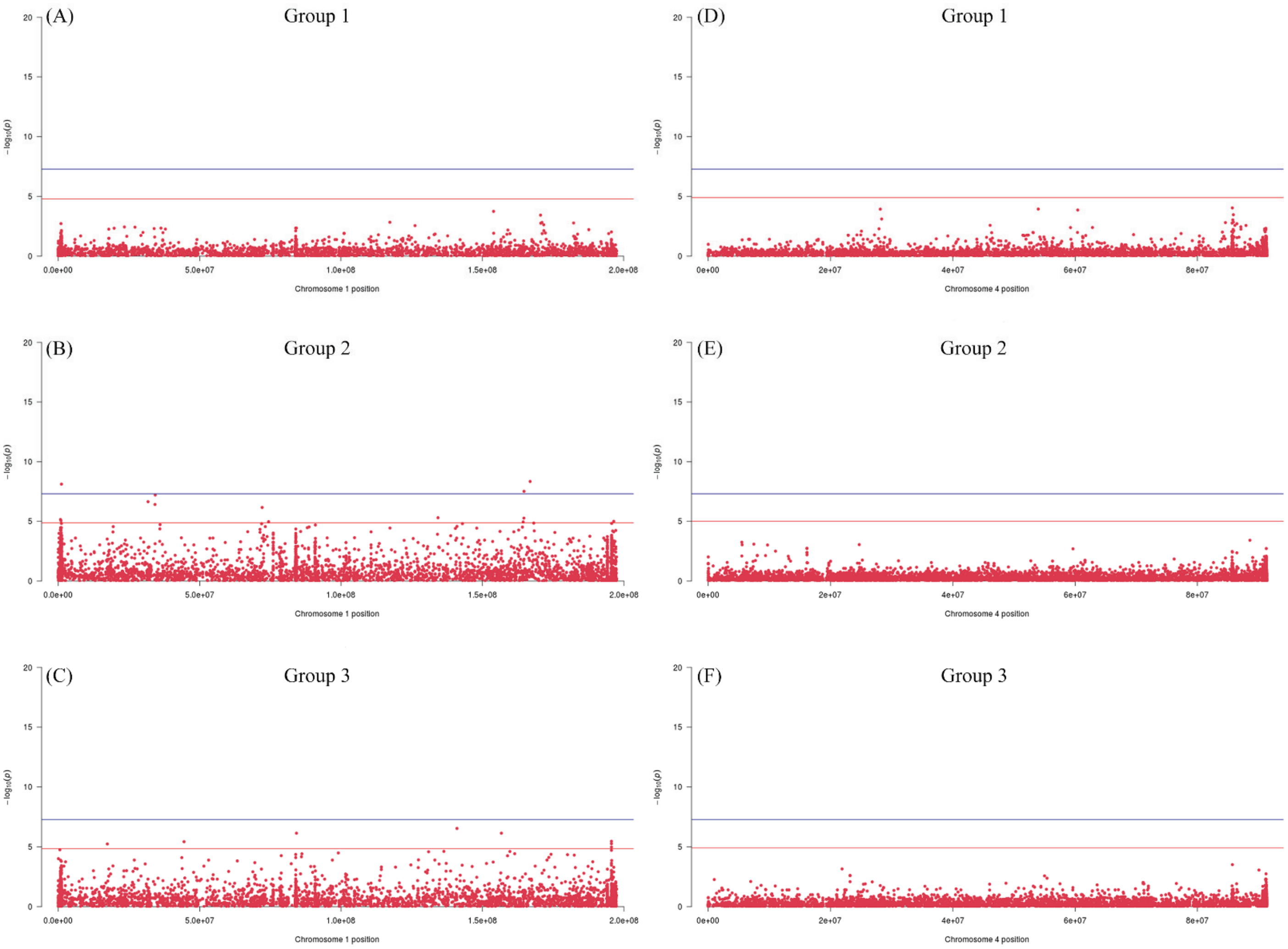

Figure S2 The association studies of structural variations for three groups on GGA1 and GGA4 respectively. The red horizontal line shows the Bonferroni threshold of P-value (0.05/the number of tested variants), and a suggestive cut-off threshold ($P=5x10^{-8}$) is indicated by blue horizontal line. To investigate the variation associated with the bantam phenotype, we tested the Structural Variation (SV) in the dataset. Firstly, we checked the variation within the genetic region that is of high risk identified by SNP based GWAS. We do not have the evidence that any deletion or duplication in *HMGA2* gene is associated with bantam in the three bantam groups. The associated variation was also manually checked for coverage as well. Secondly, using the information of the genotype of each variation, we conducted an association study in each group. In group 2, a duplication spanning GGA1:1,101,992-1,102,562 shows tentative association with the bantam phenotype, the frequency of the duplication in case:control is 44%:14%. The duplication is in the first intron of *ARF5 (ADP-ribosylation factor 5)*. However, it is not clear that *ARF* may functionally influence body growth. Two deletions, GGA1:164,677,342-164,677,819, and GGA1:166,849,859-166,849,983, are additionally reported as association, however there is no gene annotated to the deletions.

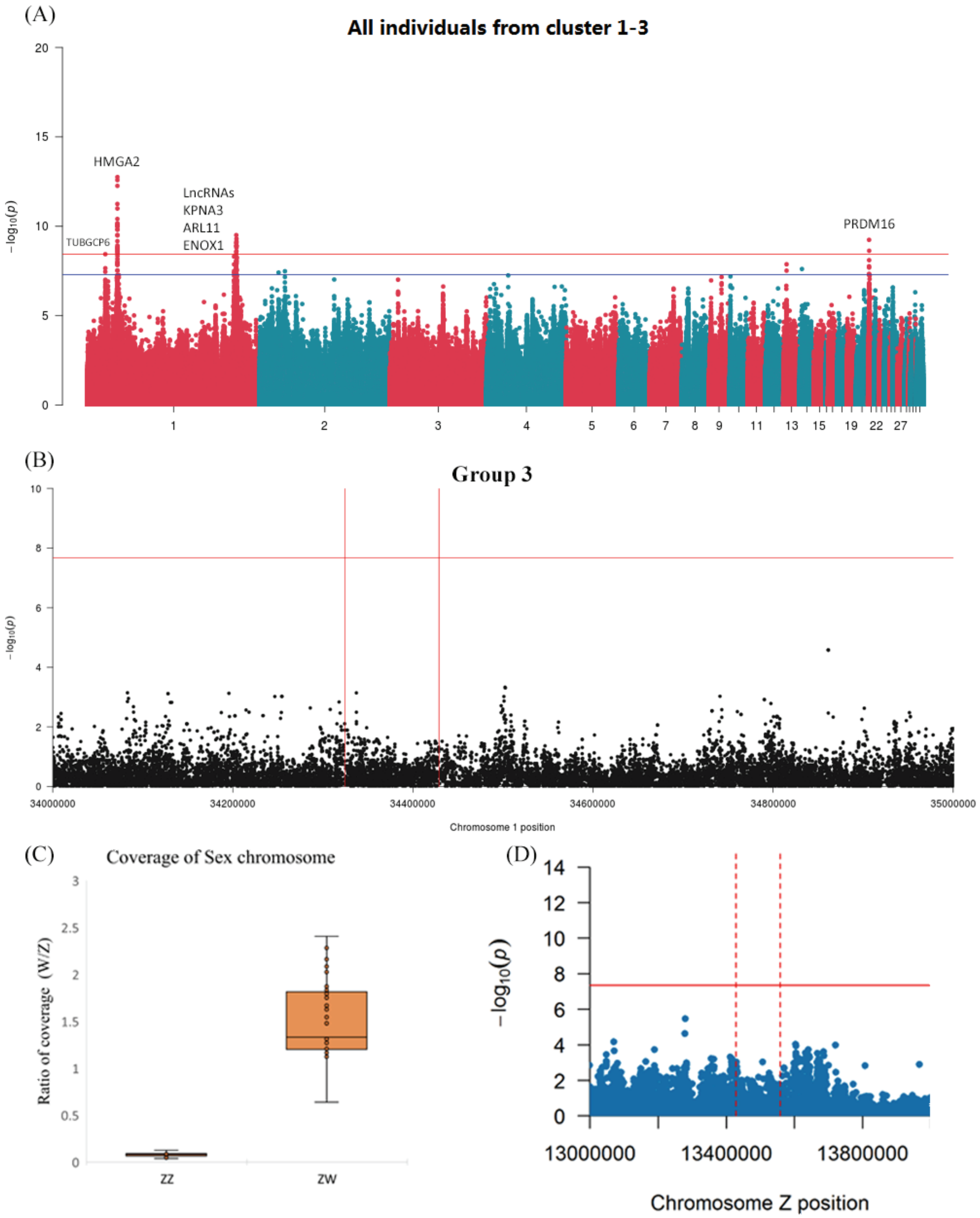

Figure S3 Manhattan plot of GWAS results of the "pooled" analysis, HMGA2 analysis in group 3 and sex-linked analysis. (A) Manhattan plot of "pooled" GWAS using all the individuals in the dataset (group 1,2, and 3). This GWAS is pooling all breeds from the three groups together, the red horizontal line shows the Bonferroni threshold, and a conventional cut-off value ($P=5x10^{-8}$) is indicated by blue horizontal line. We found some suggestive peaks and the related genes are denoted with gene symbols. (B) Regional Manhattan plot shows the HMGA2 interval in group 3. The figure shows the zoomed in chromosomal region around GGA1:34-35Mb, the red vertical lines denote the interval commonly found in group 1 and 2. (C) The coverage of sex chromosomes (Z and W). The y-axis shows the ratio of coverage between the sex chromosomes(W/Z). The inferred sex of individual is displayed at x-axis. (D) The Manhattan plot of meta-analysis integrating the results from the three group shows the association around GHR gene. The red vertical lines show the location of GHR gene (GGAZ:13.43-13.55Mb).

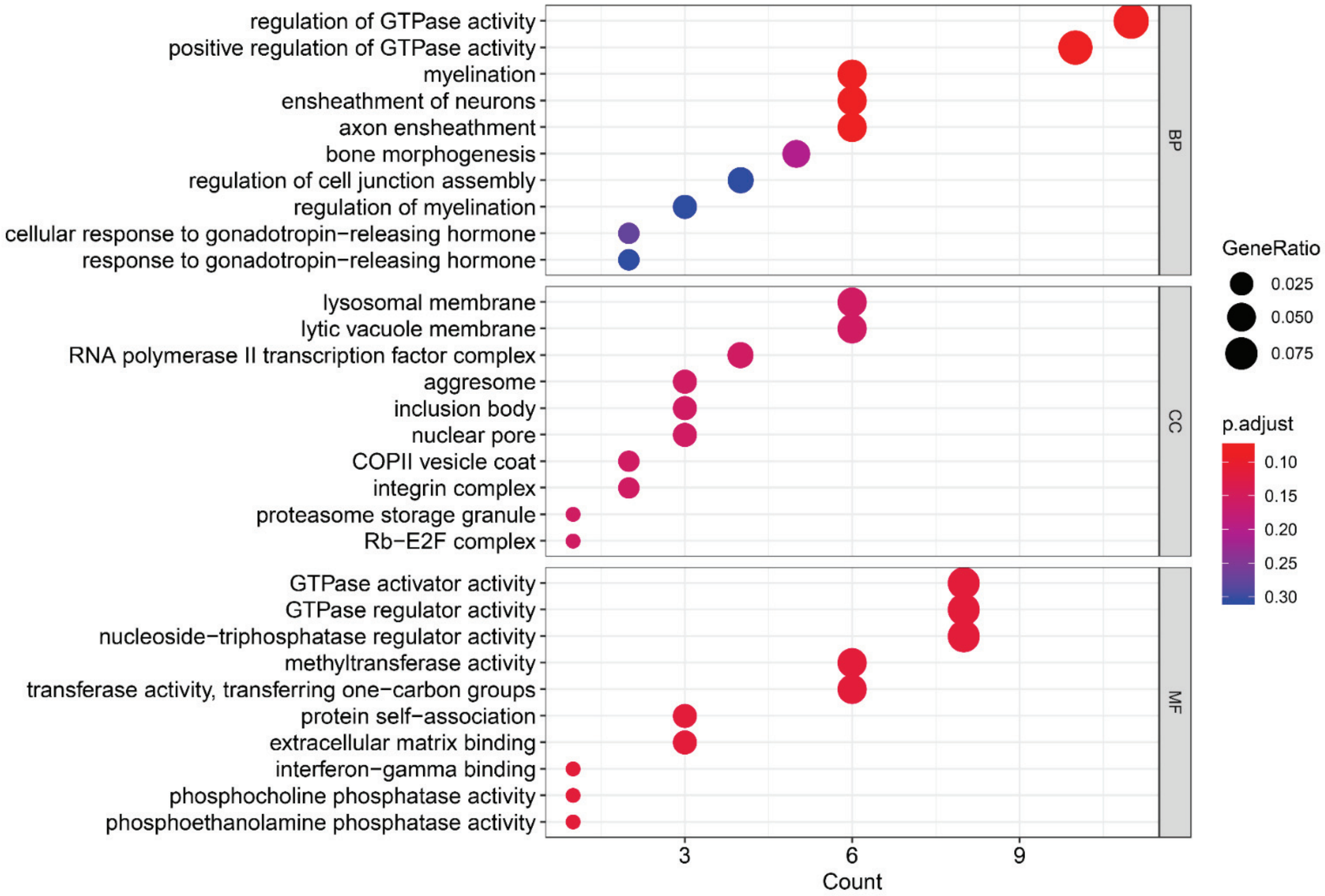

Figure S4 The enrichment analysis of Gene Ontology (GO) terms. The gene set includes associated genes identified by four studies (the three group-based GWAS and the meta-analysis). Three GO categories, BP, CC and MF are shown by the three plots, with colored circle as the adjusted P value, the x-axis as the count of the terms, and the size of the circle as the gene ratio. We have found no statistically significant enrichment, but the GO terms associated with bone morphogenesis, developmental process, skeleton muscle, and bone development can be found in Table S6.

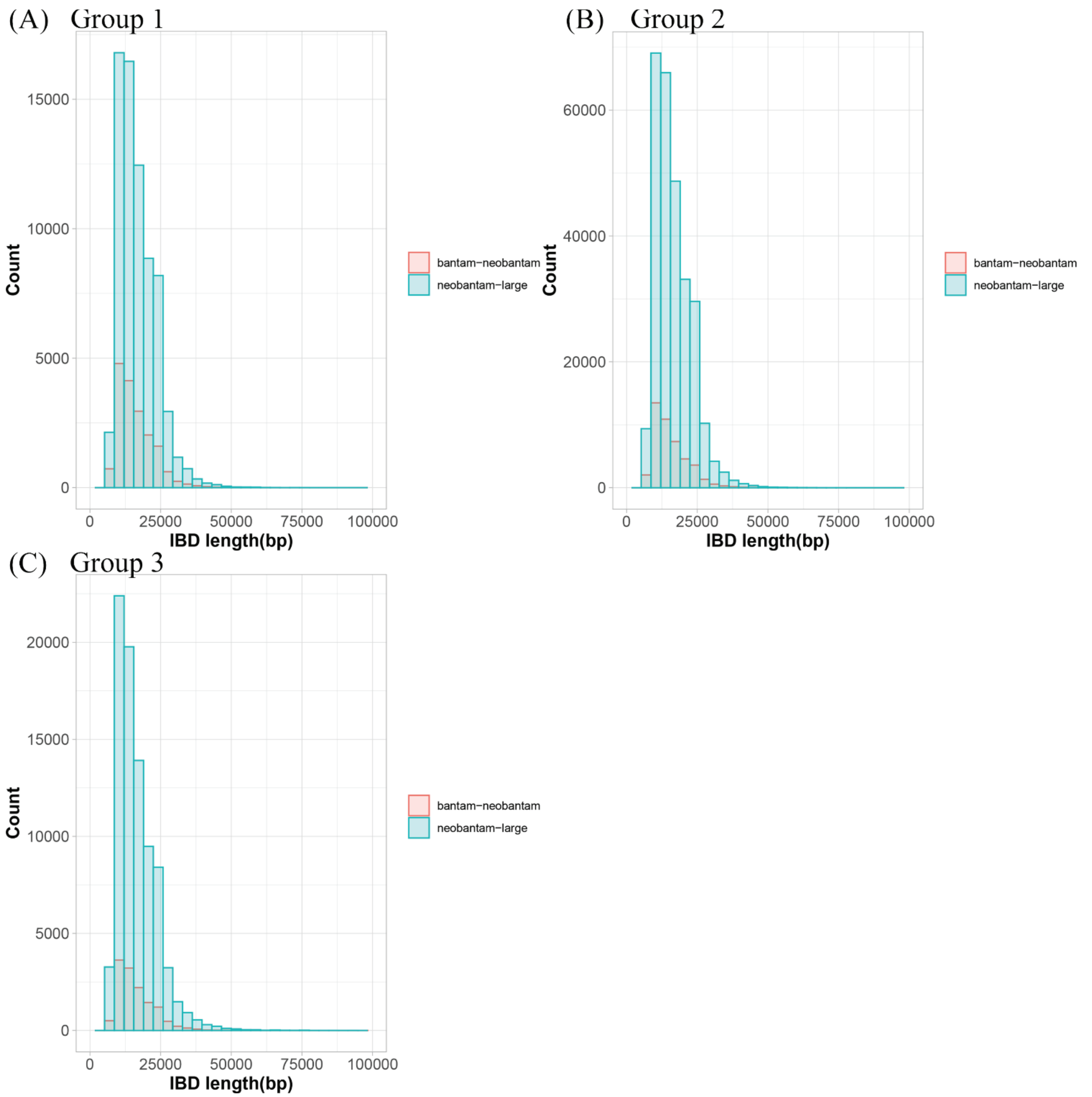

Figure S5 The histogram shows the comparison of the count of IBD fragments in the three groups. In each group, the count of IBD on GGA1 (at length in bp) between bantam and neo-bantam is plotted (denoted as bantam-neobantam in red), as well as the IBD between neo-bantam and their normal-sized counterpart (denoted as neo-bantam-large in green).

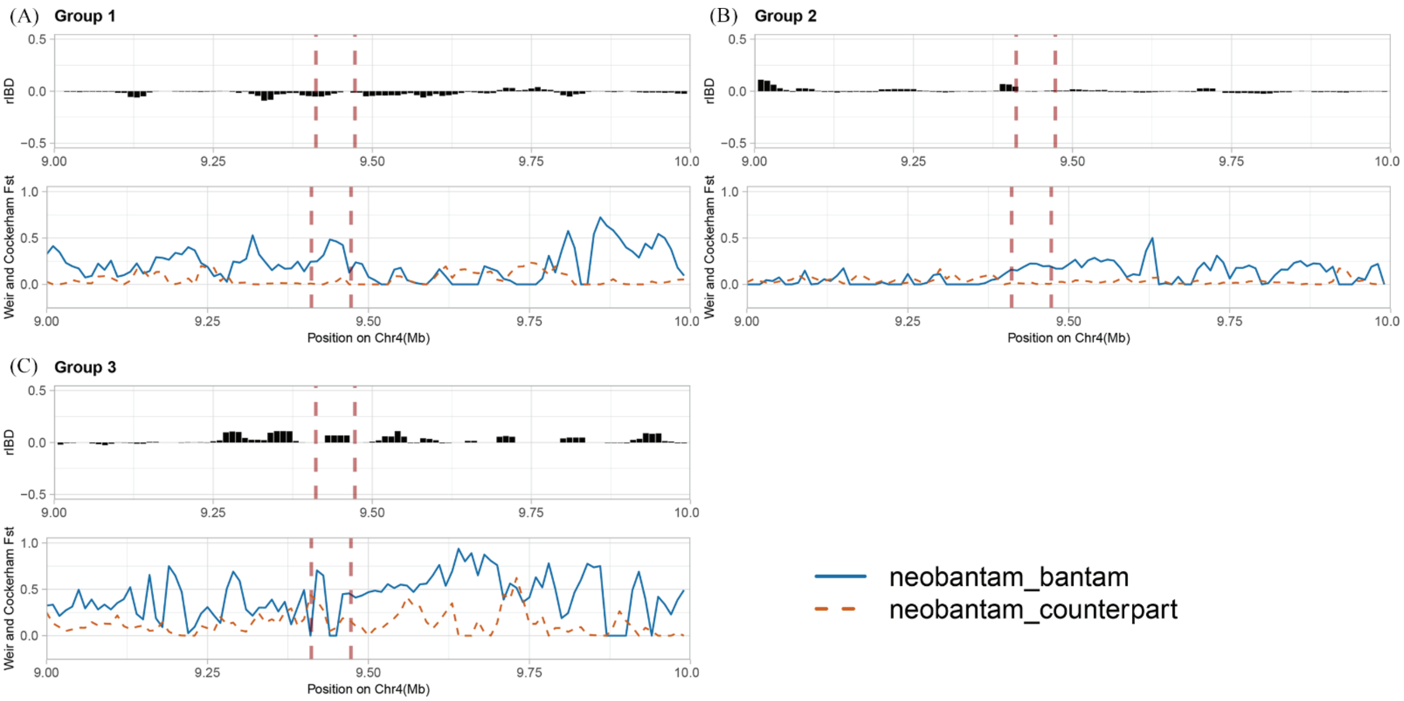

Figure S6 Distribution of rIBD and Fst estimation on GGA 4. The group 3 associated region on chromosome 4 was shown. The dashed lines indicate the interval on chromosome 4. In each group, the upper panel shows the estimation of rIBD, and the lower panel displays the Fst estimation.

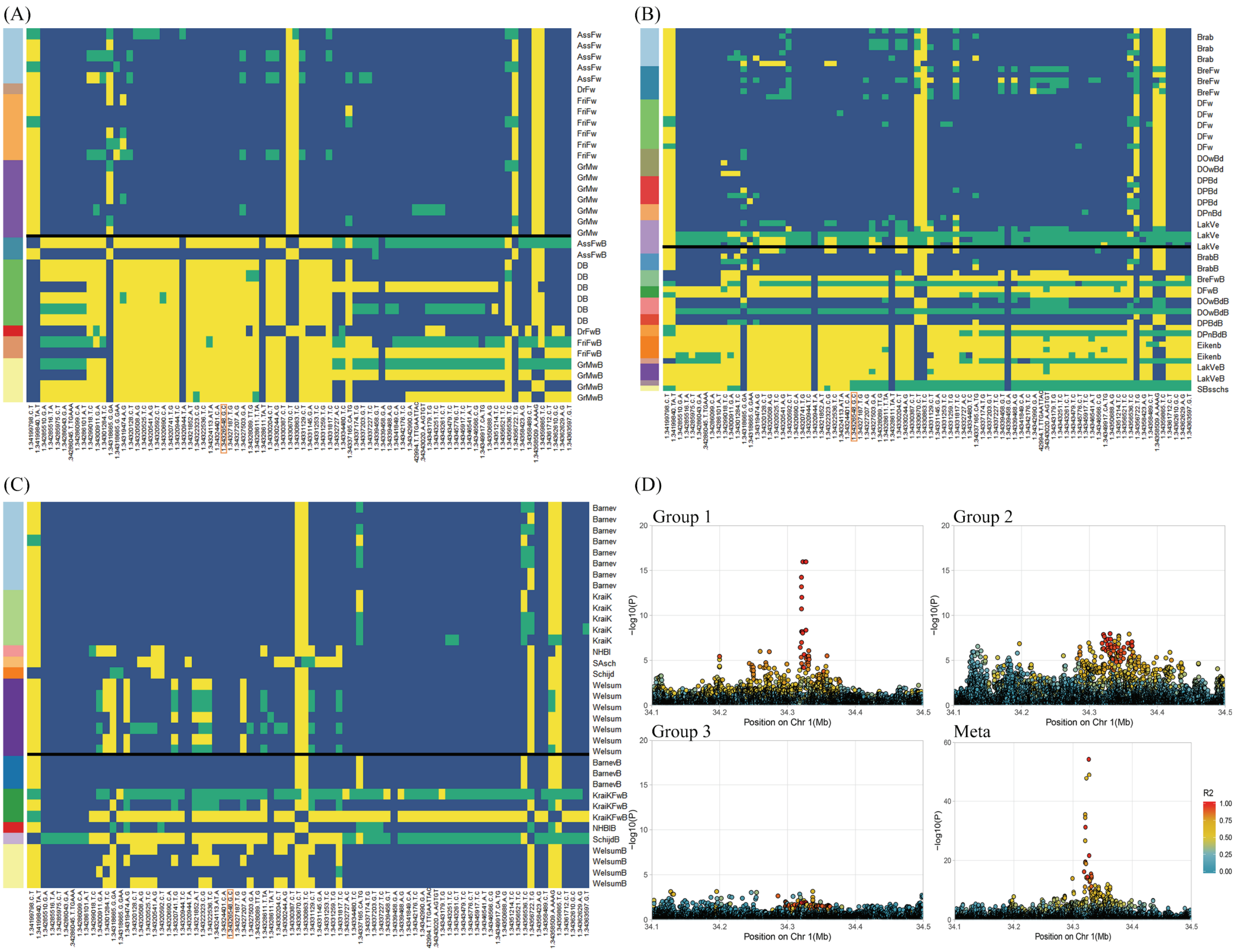

Figure S7 The haplotype of genomic associated sites in three groups. (A-C) The horizontal axis represents the significant variant sites surrounding GGA1:34-35Mb ordered by chromosome and position, each row represents one individual, the breed name is on the right vertical axis and annotated by the left colored boxes. The bantam and normal-sized breeds are separated by a black horizontal line. The highlighted SNP in the orange box on the horizontal axis is the most significant peak variant (NC_006088.5:g.34326548G>C) found in the meta-analysis. (D) Manhattan plot shows the computed linkage disequilibrium (r^2) between markers in the three groups (group 1 to 3) and meta-analysis. The value of r^2 was calculated for each variant against the peak SNP (NC_006088.5:g.34326548G>C). The colors of points indicate the values of r^2, ranging from 0 (blue) to 1 (red).

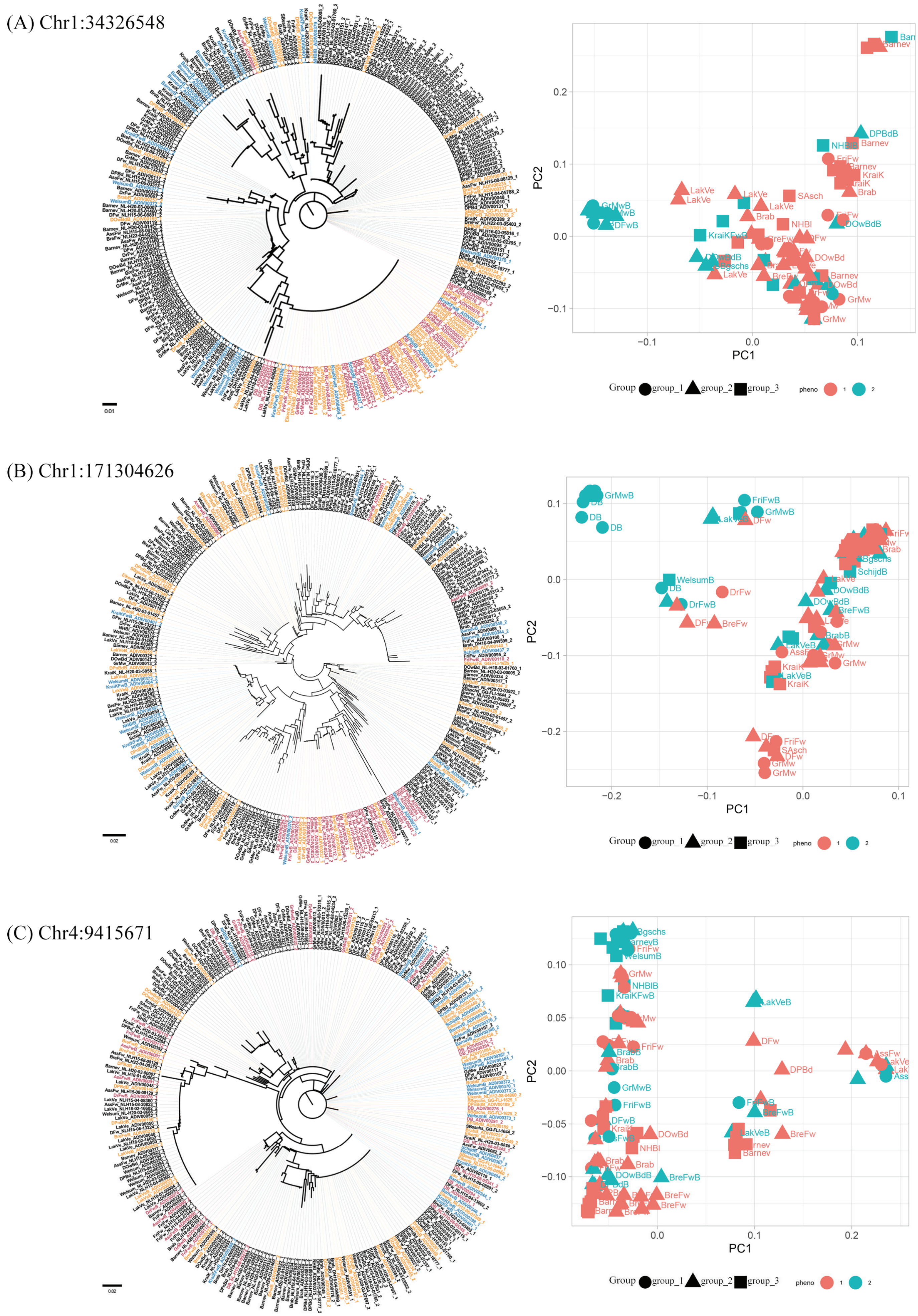

(A) Chr1:34326548
PC1
PC2
Group group_1 group_2 group_3
pheno 1 2
(B) Chr1:171304626
PC1
PC2
Group group_1 group_2 group_3
pheno 1 2
(C) Chr4:9415671
PC1
PC2
Group group_1 group_2 group_3
pheno 1 2

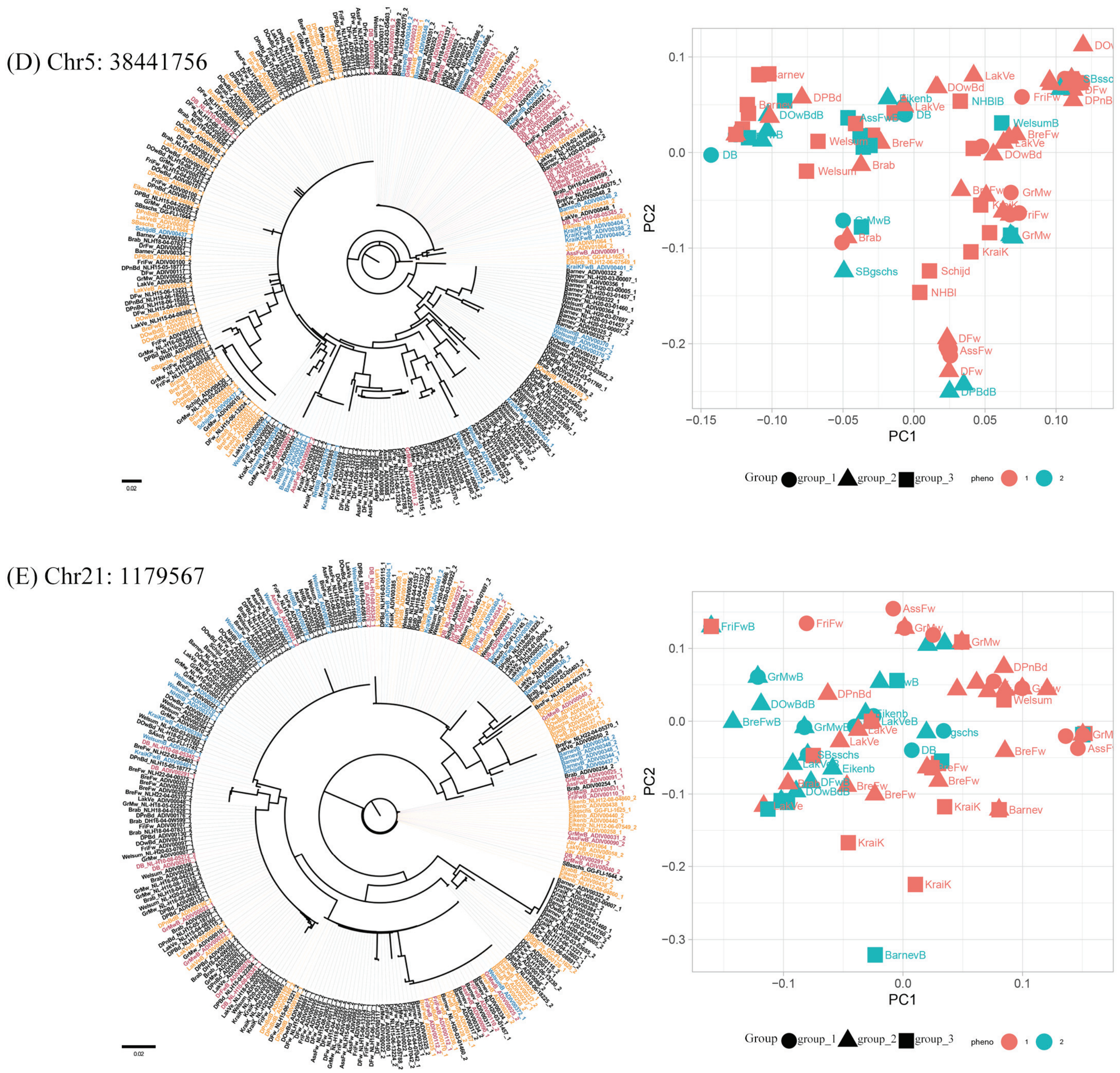

Figure S8 NJ-tree topology of haplotype and PCA structure. In the NJ-tree panel, we used the haplotype coves the variant of interest and 1Kb surrounding region. The two haplotypes of one individual is denoetd by the number one and two at the end of the tip name. The color scheme is based on the group, red shows bantams in group 1, yellow shows bantams in group2, and bantams in group 3 are in blue nodes, and the normal-sized ones are colored black. In the PCA panel, the phenotypes (1 is for normal-sized, 2 is for bantam) are shown in colors, and groups are shown in different shapes. We showed 5 haplotype blocks, denoted by the lead variants: (A) Variant NC_006088.5:g.34326548G>C; (B) SNP (rs732144338) has the frequency of bantam associated allele in case:control of each group as, 45.67%: 0.00% (group 1); 10%: 2.5% (group 2), while in group 3 it is not tested in this study. (C) SNP (rs13643124) has the bantam allele frequency in case:control of each group as, 26.67%: 23.68% (group 1); 42%: 9.76% (group 2); 80.67%: 5.83% (group 3). (D) SNP (rs316652476) has the bantam allele frequency as, 90%: 2.78% (group 1); 20%:19.51% (group 2); 61.11%: 31.82% (group 3). (E) Variant (rs735861847) has the bantam allele frequency in case:control 75%: 26.32%(group 1); 79.17%: 10% (group 2); 38.89%: 36.36% (group 3). Specially, individuals without genotype in this interval were removed in the PCA, the imputed haplotype was used in phylogeney construction. The position shown in the figure is based on the genome build GRCg6a (GenBank Accession: GCA_000002315.5).
